# Two Coupled Continuous‐Flow Ventricular Assist Devices as a Novel BIVAD With One Driveline: Acute Animal Study Results

**DOI:** 10.1111/aor.14933

**Published:** 2024-12-20

**Authors:** Sara Knigge, Günes Dogan, Ezin Deniz, Jasmin Hanke, Ali Merzah, Dominik Berliner, Fanwu Kong, Torsten Heilmann, Bastian Schmack, Aron F. Popov, Alexander Weymann, Johann Bauersachs, Arjang Ruhparwar, Jan D. Schmitto

**Affiliations:** ^1^ Department of Cardiac‐, Thoracic‐ Transplantation and Vascular Surgery, Hannover Medical School Hannover Germany; ^2^ Department of Cardiology Hannover Medical School Hannover Germany; ^3^ HXCORMED Shenzhen China; ^4^ ReCO2very Therapies GmbH Bad Klosterlausnitz Germany

**Keywords:** acute animal model, biventricular support, DuoCor BiVAS

## Abstract

**Background:**

The study assesses the feasibility of the DuoCor BiVAS, a novel biventricular assist system integrating magnetic levitation technology.

**Methods:**

In an acute large animal model involving five sheep, each received the DuoCor BiVAS without cardiopulmonary bypass. Hemodynamic and device parameters were monitored continuously for 1‐h post‐implantation.

**Results:**

Intraoperative implantation was uneventful, demonstrating successful biventricular support with mean blood flows of 5.4 LPM (left) and 5.5 LPM (right). Analysis showed proportional flow rates and relationships between pump speed, flow, and power consumption. No adverse events like thrombus formation, bleeding, stroke, or device failure occurred.

**Conclusion:**

This research underscores the DuoCor BiVAS's potential for severe biventricular heart failure treatment, providing insights into its feasibility and functionality in acute animal models. The findings suggest promising clinical applications, particularly with the system's single driveline design potentially enhancing patient mobilization and quality of life. Further investigations are needed to advance this technology for broader clinical use.

## Graphical abstract

The DuoCor BiVAS, a novel biventricular assist system, demonstrated successful implantation and stable hemodynamic support in an acute large animal model. The study highlights the system's potential for treating severe heart failure, with promising implications for patient mobilization and quality of life, warranting further clinical investigation.

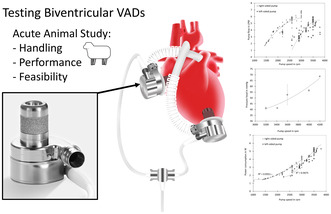

**Abbreviations**
ACT: activated clotting time
BiVAS: biventricular assist system
HLM: heart‐lung machine
LAVES: Niedersächsisches Landesamt für Verbraucherschutz und Lebensmittelsicherheit
LV: left ventricular
LVAD: left ventricular assist device
PI: Pulsatility Index
TAH: total artificial heart

## 1 Introduction

Heart failure rates are rising in developed countries, making advanced treatments like left ventricular assist device (LVAD) implantation or heart transplantation critical for patients with severe left ventricular (LV) failure [1, 2, 3, 4, 5]. LVADs serve as vital support for those awaiting a transplant or with no viable transplant options, especially for patients with low LV ejection fraction who are at high risk of early mortality. However, for those facing both left and right heart failure, treatment options are limited. Biventricular support options include extracorporeal assist systems, such as the Excor Mobile (BerlinHeart, Germany), which require a large, trolley‐bound air compressor. Total artificial hearts (TAHs), like Aeson (CARMAT, France), offer an alternative but are more invasive and are only indicated for a minority of patients who have completely lost heart function.

One emerging approach to address biventricular failure is the off‐label use of two HeartMate 3 LVADs (Abbott, Illinois, United States) on both sides of the heart. This method has been trialed in configurations as a biventricular assist system (BiVAS) [6, 7, 8] or as a continuous‐flow TAH [7]. Recent studies indicate that patients with significant LV and right ventricular failure have benefited from the BiVAS approach, which provides greater mobility and quality of life compared to extracorporeal systems. A multicenter study involving 14 patients demonstrated the feasibility and success of fully magnetically levitated centrifugal BiVAS support, with encouraging results over extended support durations [9].

Nevertheless, dual LVAD configurations have notable drawbacks, including the need for two drivelines, which doubles the infection risk, and two separate controllers and battery systems, which place high demands on patients. These limitations arise because the HeartMate 3 was not designed for biventricular support, necessitating the use of two independent devices for each ventricle [10, 11].

To address these issues, our study explores the DuoCor BiVAS, a single, integrated device developed by HXCORMED. The DuoCor system features two pumps connected by a single driveline to a single controller, with only two small batteries. This streamlined design aims to improve patient mobility, ease of use, and reliability. The device leverages advanced magnetic levitation technology to offer effective biventricular support with enhanced convenience for both patients and healthcare providers.

Our study investigates the feasibility of the DuoCor BiVAS through an acute animal model study on sheep. We focus on surgical implantation procedures, device performance in terms of flow dynamics and power efficiency, controller software functionality, and overall usability. The findings will provide valuable insights for device developers on design, handling, and control, contributing to the evolving field of mechanical circulatory support.

## 2 Material and Methods

### 2.1 Device

**FIGURE 1:**
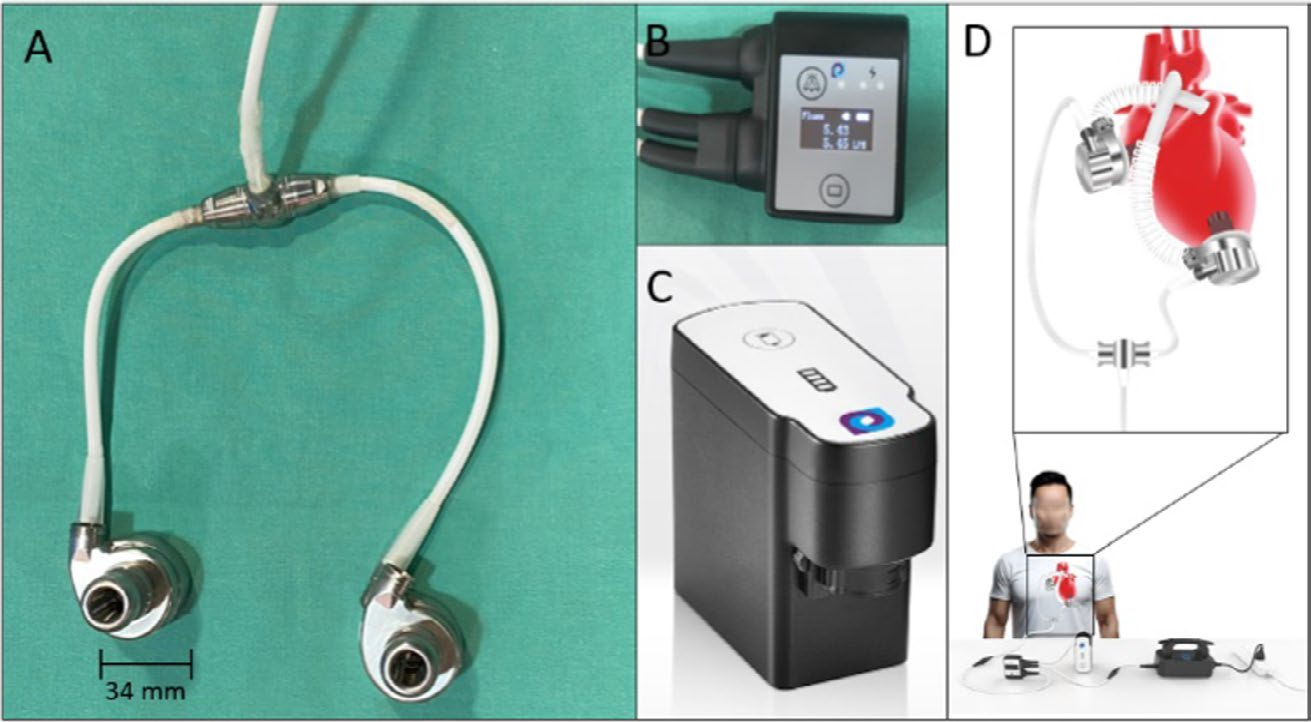
DuoCor BiVAS, including two coupled pumps (A), one controller (B), battery (C), and schematic picture of a patient with an implanted DuoCor BiVAS, intracorporeal T‐connector and its peripheral devices such as the controller, one battery, and its power supply unit (D). [Color figure can be viewed at wileyonlinelibrary.com]

The novel DuoCor BiVAS consists of two coupled pumps, one for the left ventricle and one for the right ventricle, simultaneously pumping blood to the systemic and pulmonary circulations. The weight of the DuoCor BiVAS is 204 g. Each pump's ventricular‐facing portion measures 34 mm in diameter and 26 mm in thickness. The pumps' inlets are sintered to facilitate the endothelial tissue ingrow on blood‐contacting surfaces. Alongside the pumps, the system includes one single control unit, which controls both pumps, as well as two compact batteries totaling a weight of 800 g. The two pumps are identical and connected to the controller by a single driveline that splits up in the patient's body using an intracorporeal T‐connector. Figure 1A–D depicts the components of the DuoCor BiVAS and their assembly.

### 2.2 Animal Experiment

The experimental study aimed to evaluate the feasibility, safety, and efficacy of the DuoCor BiVAS. Five female blackface sheep weighing between 69 and 87 kg were utilized in this study under sedation (2–3 vol% Isofluran; Forane Servopharma GmbH, Germany in oxygen/air (2:1)), and continuous monitoring. Anticoagulation was maintained using 400 I.U./kg(body weight) unfractionated heparin (Clexane, Sanofi‐Aventis, Germany) (activated clotting time [ACT]> 190 s). Arterial blood pressure was monitored in the carotid artery with a catheter. All animals were hemodynamically stabilized using a heart‐lung machine (HLM) while the pumps were surgically implanted.

The BiVAS pumps were integrated into the sheep's circulatory system following a standard open‐heart surgery procedure. Specifically, this process involved establishing distinct connections for each pump with sewing rings. One pump was surgically implanted on the left side of the heart, positioned at the LV apex with the inflow cannula in parallel to the ventricular septum and toward the mitral valve, followed by an outflow graft connection with end‐to‐side anastomosis to the ascending aorta. Simultaneously, the second pump was implanted on the right side of the heart, with the inlet strategically placed within the right atrium and the outflow graft directed end‐to‐side into the pulmonary artery. After the onset of the coupled pumps, the function of the HLM was reduced stepwise until the BiVAS fully achieved circulation. Once the BiVAS achieved total cardiac output, the HLM was stopped, HLM cannulas were explanted, and the animals were followed up for the next hour to investigate the hemodynamic function of both pumps.

The pump's log file was used to analyze pump efficiency and performance regarding power consumption, dependence of pump speed and flow rate, and flow matching of the left‐sided and right‐sided pumps.

All animal experiments were approved by the local ethical committee, in Hannover, Lower Saxony, Germany.

### 2.3 Data

Continuous data were presented as individual data points. Graphs were analyzed to identify regression curves, and the quality of these curves was assessed using goodness‐of‐fit values. All calculations were conducted in Microsoft Excel 2019 (Microsoft Corporation, USA).

### 2.4 Statement of Ethics and Humane Animal Care

This study complies with the ethics statement. All animal procedures were conducted in accordance with the ethical standards and guidelines set forth by Nds. Landesamt für Verbraucherschutz und Lebensmittelsicherheit (LAVES). The protocols were designed to minimize animal suffering and ensure humane treatment throughout the study. Before commencing the research, approval was obtained from the LAVES under protocol number 23‐00422.

## 3 Results

### 3.1 Surgical Procedure

During the acute experiment, five animals underwent surgical procedures. The duration of the device implantation was approximately 4–5 h in all animals, involving 1 h of preparation for implantation and approximately 2 h dedicated to the actual procedure as well as control of bleeding and hemodynamics.

The anastomosis to the aorta and pulmonary artery typically consumes 15–20 min. Following this, on average, the fixation‐cuff implantation on the right atrium and the LV apex takes approximately 10–15 min each. Subsequently, the pump implantation, including positioning and adjustment, requires an average of 5 min. The surgical instruments supplied with the device proved valuable and well‐designed. However, after some use, surgeons provided feedback suggesting additional improvements for enhanced handling, for example, one suggestion about the pump component, where a recommendation was made to opt for a softer cuff material. This adjustment aimed to facilitate sewing and improve the device's ability to cling to the patient's tissue more effectively. Another suggestion was to adjust the lever closure that connects the pump to the cuff. The surgeons expressed a desire for a more pronounced haptic feedback of the “click” to clearly indicate the correct closure.

**FIGURE 2:**
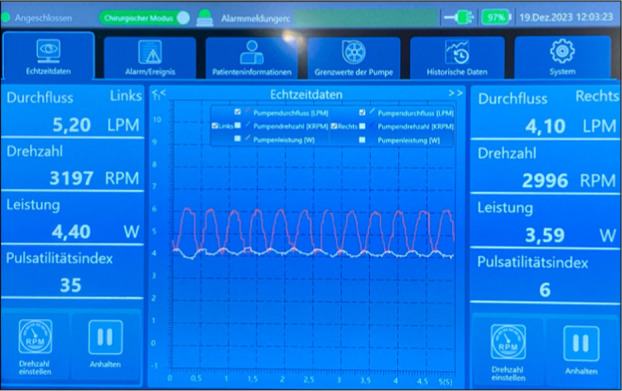
User interface for surgeons and caretakers demonstrating flow‐rate adjustment for left and right sides, essential for evaluating pump performance, alongside a time diagram of various parameters (language mode: German). [Color figure can be viewed at wileyonlinelibrary.com]

**FIGURE 3:**
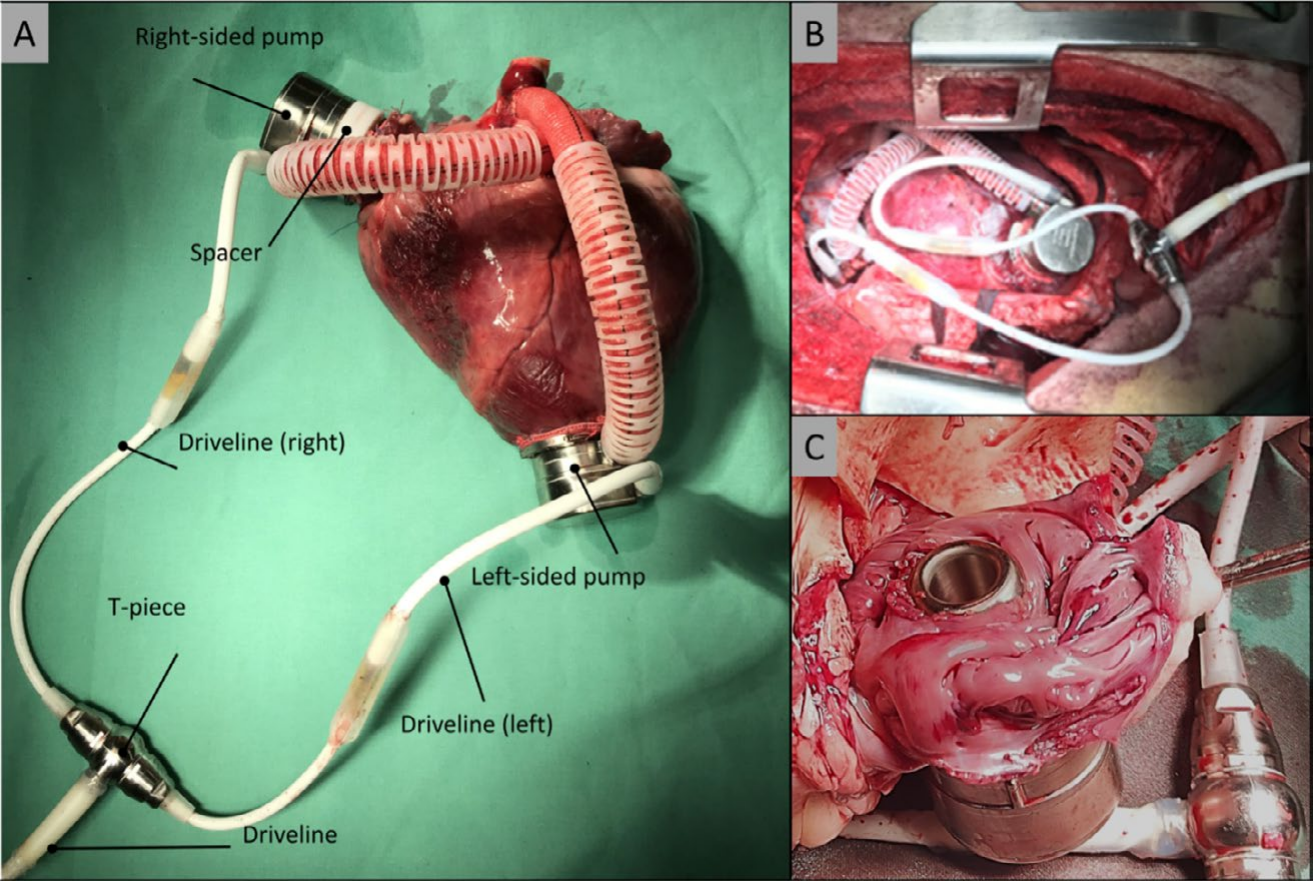
Explanted sheep heart showing the DuoCor BiVAS implanted at the left ventricular apex and right atrium. Both drivelines are connected to a single T‐connector, forming one driveline that can be tunneled outward through the abdomen (exiting driveline). (A); DuoCor BiVAS in situ within the chest cavity (B); View of the inner wall of the right atrium (C). [Color figure can be viewed at wileyonlinelibrary.com]

Only minor suggestions became necessary for further improvement of the user interface. The focus remained on intuitiveness, including changing some button placements on the screen. In Figure 2, the surgical mode of the interface is depicted, showcasing the method for adjusting flow rates on the left and right sides of the device. Specific silicon spacers, another innovation by the company's engineers, allow for different depths of inflow cannula insertions into the heart's cavities. The spacers were employed to ensure the precise positioning of the right‐sided pump within the right atrium (see Figure 3A). These spacers maintain the appropriate alignment of the pump inlet within the inner wall of the atrium, ensuring that the pump's inlet remains at the perfect level (see Figure 3C). By ideal adjustment of the depth of the inflow cannula, suction events can be prevented and thromboembolic events can be reduced.

In the five completed BiVAS implantations, the DuoCor devices exhibited excellent and stable functionality without any untoward incidents in the sheep. The BiVAS effectively supplied the animals with a blood flow of 4.5–5.5 LPM through both pumps of the DuoCor BiVAS.

Table 1 lists the sheep's blood pressure before implantation and with running DuoCor BiVAS. Although the hemodynamics of all sheep were slightly below the expected physiological range, no complications were observed, meaning no thrombi, bleeding, stroke, or device failure was observed. The effects of anesthesia most likely cause the perioperative hypotension. This aspect should be addressed in chronic studies by thoroughly monitoring blood pressure in a stable, awake condition.

**TABLE 1:** Systolic and diastolic blood pressure values and pulsatility index (PI) of the sheep.

	Arterial blood pressure (Sys/Dia)		PI right side	PI left side
Sheep	Before implantation	Running BiVAS (4.5 LPM)		
1	84/55	70/66	4–5	30–40
2	90/51	72/68	5–6	30–40
3	69/43	73/71	4–5	35–35
4	71/44	68/56	4–6	30–40
5	88/45	78/65	5–6	32–38
6	78/ 44	70/61	4–5	32–36

Infrequent suction events of the left‐sided pumps were noted but promptly resolved by adjusting the pump speed. Furthermore, based on the low pulsation power of the right atrium, the Pulsatility Index (PI) on the right side was mainly permanently lower than 5 in all sheep. It remained stable within the desired range of < 50 on the left side.

### 3.2 Pump Performance

**FIGURE 4:**
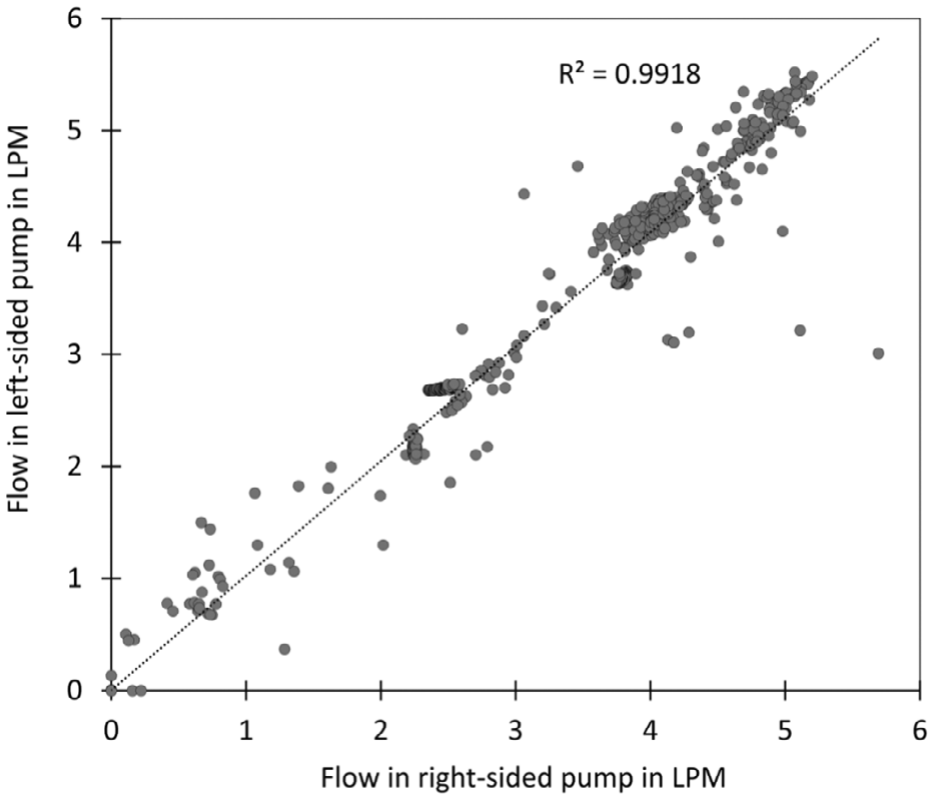
Correlation between the right and left pump flows of the DuoCor BiVAS system over a 1‐h and 17‐min period, as collected from a log file.

Figure 4 shows that both the left‐ and right‐sided pumps provide equivalent volumetric flow. This observation is supported by a linear relationship between the flow rates, which is directly proportional, with a proportionality constant of 1.02. Additionally, the correlation coefficient of 0.99 underscores the strong association between the flow rates generated by the left‐ and right‐sided pump.

**FIGURE 5:**
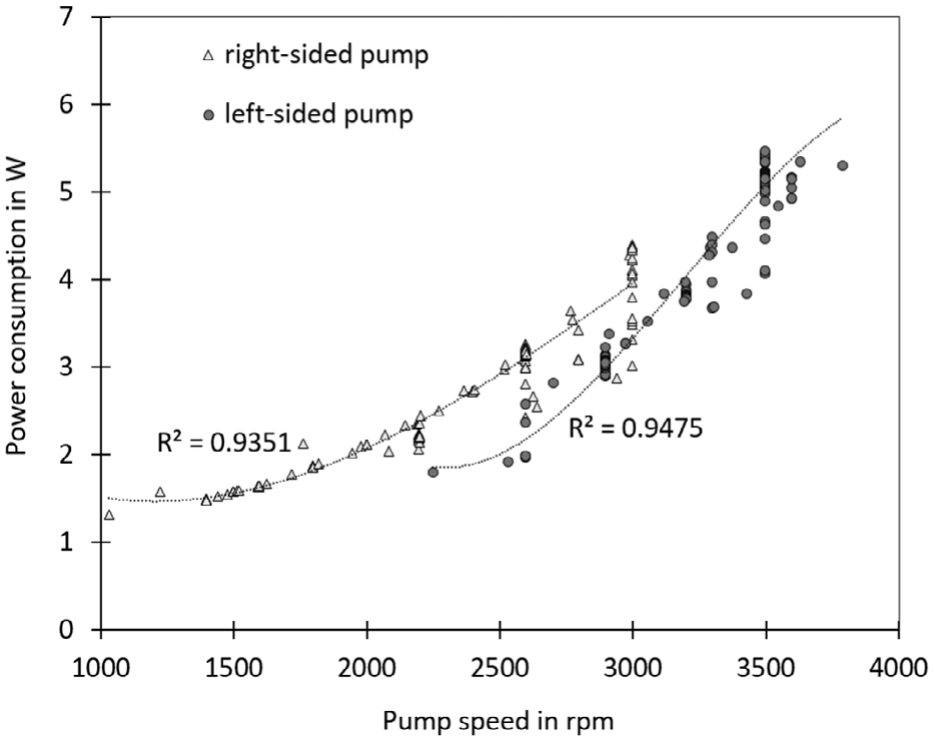
Correlation between pump speed and power over a 1‐h and 17‐min period, as collected from a log file.

The analysis of the log files in Figure 5 revealed a direct relationship between power consumption and pump speed on the left and right sides. The aortic pressure was measured at 60–80 mmHg, and the central venous pressure was held at 10 mmHg. The pump performances were well‐described by polymeric (cubic) fitting, with *R* ^2^ values of 0.94 for the right‐sided pump and 0.95 for the left‐sided pump. This behavior aligns with the typical characteristics of centrifugal pumps, in which the power consumption is proportional to the cube of the pump speed (power consumption ~ pump speed [3]). Since power consumption is highly dependent on the blood pressure in the outflow graft, low blood pressure can lead to an underestimation of power consumption. This factor should be investigated further in chronic animal trials and in vitro studies. The reported acute animal trial does not provide insights into pump performance during ambulatory conditions or physical activity.

**FIGURE 6:**
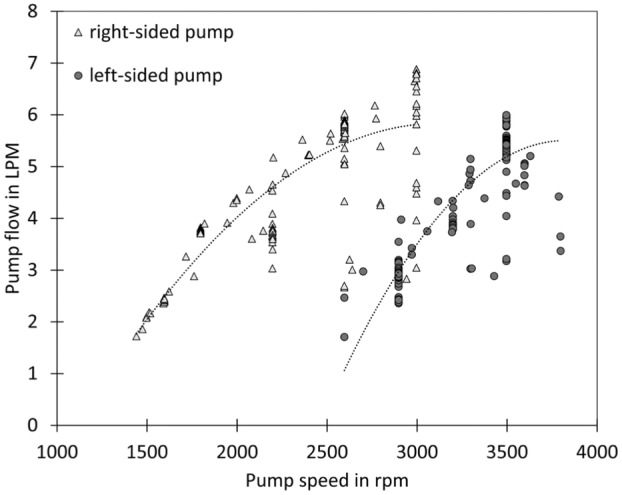
Relationship between pump speed and pump flow.

Figure 6 shows the dependency between pump speed and pump flow. As pump speed increases, the pump's flow rate also increases. The left‐sided pump curve is shifted to the right side, meaning that flow rates can only be reached at a higher pump speed. In centrifugal pumps, the flow rate is heavily influenced by the pressure difference between the inlet and outlet. This influence explains why a higher pump speed is required to achieve the same flow rate in the left‐sided pump compared to the right‐sided pump. The relationship between pump speed and flow rate exhibits a nonlinear dependency. This nonlinearity can be attributed to several factors. For example, changes in pressure in the aorta and the pulmonary artery are due to increased flow provided by the pumps. Additionally, losses due to friction, turbulence, or cavitation within the pump or the system can cause deviations from the ideal linear relationship. These losses can become more significant at lower flow rates and rotational speeds.

**FIGURE 7:**
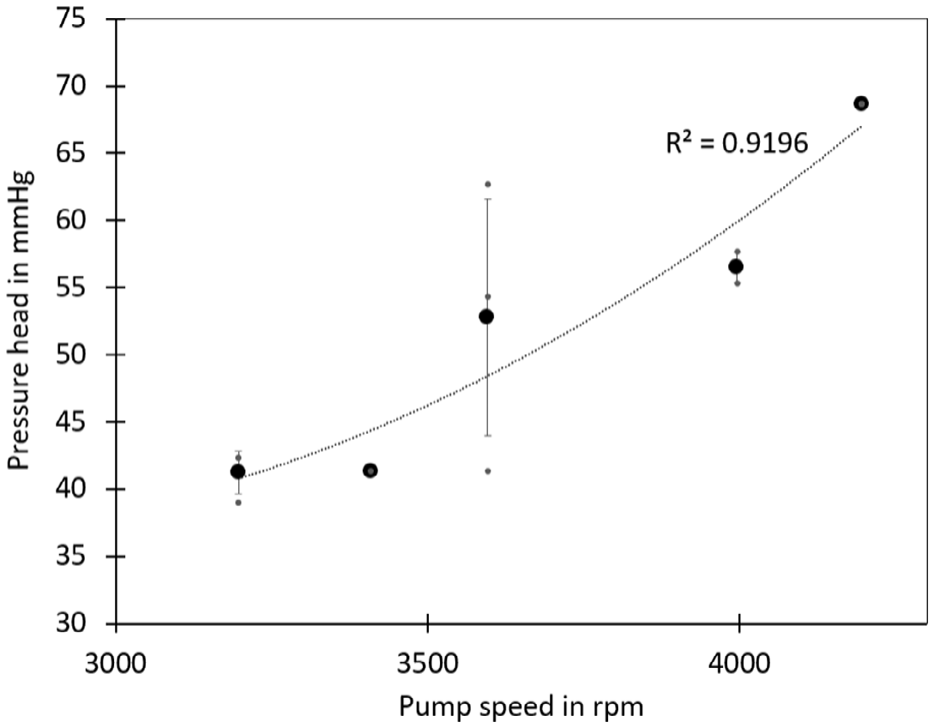
Relationship between pump speed and pressure head (Δ*P* = *P*_outlet_−*P*_inlet_) for the left‐sided pump in a sheep. The small gray points represent raw data, the black points indicate mean values, and the error bars depict the SD.

Figure 7 depicts the relationship between pump speed and pressure head (Δ*P*=*P*_outlet_‐*P*_inlet_) in the animal trail for the left‐sided pump. The pressure head is the difference between mean aortic blood pressure and the left atrium in sheep. The pressure in the left atrium is estimated to be 9 mmHg. In general, higher pump speeds and, consequently, higher flow rates lead to increased pressure at the outlet. The pressure head is quadratically related to the pump speed, as confirmed by the trend in the depicted data (Δ*P* ~ pump speed [2]).

## 4 Discussion

The acute animal model study evaluating the feasibility and efficacy of the DuoCor BiVAS (DuoCor BiVAS, HXCORMED, China) in sheep provided valuable insights into various aspects of device performance, surgical implantation technique, hemodynamic stability, as well as device characteristics. This discussion will focus on the key findings of our study and their implications.

Para−/extracorporeal displacement pumps for biventricular heart‐failing patients, like the Excor device (BerlinHeart, Berlin, Germany), have proven to be a safe option for many patients, with a 92% survival rate after 1 year, as reported by Schmack et al. [12] However, even though it is considered to be an on‐label solution, it is known to be associated with limited patient mobility, device noise, and psychological challenges. Four exit sites of cannulas also may lead to a higher risk of infections [13, 14].

The HeartMate 3 (Abbott, USA) in a BiVAS configuration has shown to be technically feasible in rare cases but still represents an off‐label application [6, 7, 8, 9] as the company had just applied for LVAD use, at least until now. The application has a clinically adverse impact because two Heartmate 3 devices necessitate two drivelines, two controllers, and a double pack of two batteries. While proven clinically to be potentially effective in exceptional cases, this setup adds complexity and weight, requiring higher patient competency for management.

In contrast, the DuoCor BiVAS could fill a critical gap for patients with severe biventricular heart failure who may not be candidates for TAH implantation or heart transplantation. The DuoCor BiVAS, with its compact design and integrated system for biventricular support, offers a patient‐friendly alternative, reducing the risks of driveline infections and the burden of device management and enhancing the overall patient experience.

The successful implantation of the DuoCor BiVAS in five sheep without any complications demonstrated the feasibility and efficacy of this device configuration. The implantation process resulted in adequate biventricular support with excellent functionality observed.

Notable aspects of the surgical procedure included the utilization of silicon spacers to maintain precise pump alignment within the right atrium, which contributed to optimizing device placement and preventing suction events. Typical VAD support for the cardiovascular system is prone to suction events because these devices have limited sensitivity to preload. Especially when both sides are supported and the Starling mechanism, which balances the flow, is diminished [15, 16, 17].

The DuoCor BiVAS uses magnetic levitation technology. In all pump parameters, the BiVAS exhibits predictable behavior that can be described by the mathematical laws governing centrifugal pumps. The animal model proved to be an effective method for testing feasibility, although there are some limitations compared to humans. The sheep were in anesthesia and in a dorsal position, resulting in relatively low blood pressure throughout the experiment compared to humans. This might affect the data, particularly regarding pump power consumption, which is likely somehow underestimated in this study. Further long‐term studies might be helpful and have already started to evaluate the long‐term safety of the DuoCor‐BiVAS.

In conclusion, the acute animal model study provided valuable preliminary data demonstrating the feasibility, efficacy, and functionality of the DuoCor BiVAS in providing biventricular support in sheep. The study outcomes contribute to ongoing efforts in refining BiVAS technology and surgical techniques, paving the promising way for clinical applications and future trials in treating patients with biventricular heart failure.

## Authors Contribution

Jan Schmitto, Günes Dogan and, Dominik Berliner conceptualized the study and provided overall project supervision. Authors Jasmin Hanke, Ali Merzah, Sara Knigge and Ezin Deniz designed the methodology and conducted data collection. Authors Torsten Heilman, Sara Knigge, Bastian Schmack, Aron F. Popov, and Alexander Weymann performed the statistical analysis and interpreted the results. Authors Jan Schmitto, Sara Knigge and Günes Dogan carried out laboratory experiments and validated the findings. Authors Sara Knigge and Torsten Heilamann prepared the initial draft of the manuscript. Authors Fanwu Kong, Arjang Ruhparwar, Johann Bauersachs and Jan Schmitto reviewed and edited the manuscript for critical intellectual content. All authors read and approved the final manuscript.

## Conflicts of Interest

The authors declare the following financial interests and relationships that might be perceived as potential sources of bias in the research: Sara Knigge has none; Günes Dogan has none; Torsten Heilmann is an employee of ReCO2very Therapies; Ali Merzah has none; Ezin Deniz has none; Jasmin Hanke has none; Dominik Berliner has none; Fanwu Kong is full‐time employed by HXCORMED and receives regular salary payments; Bastian Schmack has none; Aron F. Popov has none; Alexander Weymann has none; Johann Bauersachs has none; Arjang Ruhparwar has none; and Jan D. Schmitto has none. The authors have no other relevant financial or non‐financial conflicts of interest to disclose.

## Declaration of Generative AI and AI‐Assisted Technologies in the Writing Process

During the preparation of this work, the author(s) used ChatGPT, an AI language model developed by OpenAI, in order to assist with generating text, refining ideas, and ensuring clarity and coherence in the manuscript. After using this tool, the author(s) reviewed and edited the content as needed and take(s) full responsibility for the content of the publication.

## References

[1] A. Michaels and J. Cowger , “Patient Selection for Destination LVAD Therapy: Predicting Success in the Short and Long Term,” Current Heart Failure Reports 16, no. 5 (2019): 140–149. 31240639 10.1007/s11897-019-00434-1

[2] J. Bauersachs and S. Soltani , “Herzinsuffizienzleitlinien 2021 der ESC,” Herz 47 (2022): 12–18. 34825250 10.1007/s00059-021-05084-5

[3] J. D. Schmitto , J. S. Hanke , A. Haverich , et al., “First Implantation in Man of a New Magnetically Levitated Left Ventricular Assist Device (HeartMate III),” Journal of Heart and Lung Transplantation 34, no. 6 (2015): 858–860. 10.1016/j.healun.2015.03.001 25920932

[4] J. D. Schmitto , S. Mariani , T. Li , et al., “Five‐Year Outcomes of Patients Supported With HeartMate 3: A Single‐Centre Experience,” European Journal of Cardio‐Thoracic Surgery 59, no. 6 (2021): 1155–1163. 33585913 10.1093/ejcts/ezab018

[5] J. D. Schmitto , S. Shaw , J. Garbade , et al., “Fully Magnetically Centrifugal Left Ventricular Assist Device and Long‐Term Outcomes: The ELEVATE Registry,” European Heart Journal 45, no. 8 (2024): 613–625. 38036414 10.1093/eurheartj/ehad658 PMC10959573

[6] M. Strueber , J. D. Schmitto , I. Kutschka , and A. Haverich , “Placement of 2 Implantable Centrifugal Pumps to Serve as a Total Artificial Heart After Cardiectomy,” Journal of Thoracic and Cardiovascular Surgery 143, no. 2 (2012): 507–509. 21851956 10.1016/j.jtcvs.2011.07.034

[7] G. Dogan , J. S. Hanke , K. Alhumood , et al., “Three‐Month Outcomes After the Implantation of Two HeartMate 3 Devices in Total Artificial Heart Configuration,” Journal of Cardiovascular Surgery 64, no. 1 (2023): 121–129. 36763071 10.23736/S0021-9509.22.12445-6

[8] J.‐J. Eulert‐Grehn , P. Lanmüller , F. Schönrath , et al., “Two Implantable Continuous‐Flow Ventricular Assist Devices in a Biventricular Configuration: Technique and Results,” Interactive Cardiovascular and Thoracic Surgery 27, no. 6 (2018): 938–942. 30113626 10.1093/icvts/ivy228

[9] J. Lavee , J. Mulzer , T. Krabatsch , et al., “An International Multicenter Experience of Biventricular Support With HeartMate 3 Ventricular Assist Systems,” Journal of Heart and Lung Transplantation 37, no. 12 (2018): 1399–1402. 10.1016/j.healun.2018.08.008 30241889

[10] C. Inyom , T. Haese , F. Schoenrath , E. Potapov , and J. Knierim , “Lived Experiences of Patients Implanted With Left Ventricular Assist Devices,” Heart & Lung 55 (2022): 155–161. 35605356 10.1016/j.hrtlng.2022.05.002

[11] M. R. Mehra , D. J. Goldstein , J. C. Cleveland , et al., “Five‐Year Outcomes in Patients With Fully Magnetically Levitated vs Axial‐Flow Left Ventricular Assist Devices in the MOMENTUM 3 Randomized Trial,” Journal of the American Medical Association 328, no. 12 (2022): 1233–1242. 36074476 10.1001/jama.2022.16197 PMC9459909

[12] B. Schmack , A. Weymann , F. Ruschitzka , et al., “Successful Support of Biventricular Heart Failure Patients by New EXCOR® Adult Pumps With Bileaflet Valves: A Prospective Study,” Clinical Research in Cardiology 107 (2018): 413–420. 29294144 10.1007/s00392-017-1200-4

[13] S.‐E. Bartfay , G. Dellgren , S. Hallhagen , et al., “Durable Circulatory Support With a Paracorporeal Device as an Option for Pediatric and Adult Heart Failure Patients,” Journal of Thoracic and Cardiovascular Surgery 161, no. 4 (2021): 1453–1464.e4. 32653285 10.1016/j.jtcvs.2020.04.163

[14] J. Kremer , A. El‐Dor , R. Rivinius , et al., “Wound Infections in Adult Patients After Berlin Heart® EXCOR Biventricular Assist Device Implantation,” Life (Basel) 12, no. 10 (2022): 1550. 10.3390/life12101550 PMC9604684 36294985

[15] S. D. Gregory , M. J. Pearcy , and D. Timms , “Passive Control of a Biventricular Assist Device With Compliant Inflow Cannulae,” Artificial Organs 36, no. 8 (2012): 683–690. 22882438 10.1111/j.1525-1594.2012.01504.x

[16] M. Rocchi , C. Gross , F. Moscato , T. Schlöglhofer , B. Meyns , and L. Fresiello , “An In Vitro Model to Study Suction Events by a Ventricular Assist Device: Validation With Clinical Data,” Frontiers in Physiology 14 (2023): 1155032. 37560156 10.3389/fphys.2023.1155032 PMC10407082

[17] C. Gross , H. Schima , T. Schlöglhofer , et al., “Continuous LVAD Monitoring Reveals High Suction Rates in Clinically Stable Outpatients,” Artificial Organs 44, no. 7 (2020): E251–E262. 31945201 10.1111/aor.13638 PMC7318142

